# Clinical and prognostic implications of hyaluronic acid in patients with COVID-19 reinfection and first infection

**DOI:** 10.3389/fmicb.2024.1406581

**Published:** 2024-05-31

**Authors:** Yanyan Li, Ming Han, Xin Li

**Affiliations:** ^1^Center of Integrative Medicine, Beijing Ditan Hospital, Capital Medical University, Beijing, China; ^2^National Center for Infectious Diseases, Beijing Ditan Hospital, Capital Medical University, Beijing, China; ^3^Beijing Key Laboratory of Emerging Infectious Diseases, Institute of Infectious Diseases, Beijing Ditan Hospital, Capital Medical University, Beijing, China; ^4^Beijing Institute of Infectious Diseases, Beijing, China

**Keywords:** hyaluronic acid, severe acute respiratory syndrome coronavirus-2, coronavirus disease 2019, mortality, reinfection, first infection, long COVID

## Abstract

**Objective:**

Previous research has shown that human identical sequences of severe acute respiratory syndrome coronavirus-2 (SARS-CoV-2) promote coronavirus disease 2019 (COVID-19) progression by upregulating hyaluronic acid (HA). However, the association of HA with mortality and long COVID in SARS-CoV-2 reinfection and first infection is unclear.

**Methods:**

Patients with COVID-19 at Beijing Ditan Hospital from September 2023 to November 2023 were consecutively enrolled. SARS-CoV-2 reinfections were matched 1:2 with first infections using a nearest neighbor propensity score matching algorithm. We compared the hospital outcomes between patients with COVID-19 reinfection and first infection. The association between HA levels and mortality and long COVID in the matched cohort was analyzed.

**Results:**

The reinfection rate among COVID-19 hospitalized patients was 25.4% (62 cases). After propensity score matching, we found that reinfection was associated with a better clinical course and prognosis, including lower levels of C-reactive protein and erythrocyte sedimentation rate, fewer cases of bilateral lung infiltration and respiratory failure, and shorter viral clearance time and duration of symptoms (*p* < 0.05). HA levels were significantly higher in patients with primary infection [128.0 (90.5, 185.0) vs. 94.5 (62.0, 167.3), *p* = 0.008], those with prolonged viral clearance time [90.5 (61.5, 130.8) vs. 130.0 (95.0, 188.0), *p* < 0.001], and deceased patients [105.5 (76.8, 164.5) vs. 188.0 (118.0, 208.0), *p* = 0.002]. Further analysis showed that HA was an independent predictor of death (AUC: 0.789), and the risk of death increased by 4.435 times (OR = 5.435, 95% CI = 1.205–24.510, *p* = 0.028) in patients with high HA levels. We found that patients with HA levels above 116 ng/mL had an increased risk of death. However, the incidence of long COVID was similar in the different HA level groups (*p* > 0.05).

**Conclusion:**

Serum HA may serve as a novel biomarker for predicting COVID-19 mortality in patients with SARS-CoV-2 reinfection and first infection. However, HA levels may not be associated with long COVID.

## Introduction

When the coronavirus disease 2019 (COVID-19) pandemic emerged in early 2020, the severe acute respiratory syndrome coronavirus-2 (SARS-CoV-2) virus posed a formidable challenge, hindering global progress and plunging people’s lives into chaos (Markov et al., 2023). With the significant reduction in the pathogenicity of SARS-CoV-2, COVID-19 has become a part of a long list of common infectious respiratory diseases (Willyard, 2023). However, it is undeniable that a considerable portion of the population remains at risk of reinfection, severe infection, and death (Kim et al., 2021; Nguyen et al., 2023). The persistence of COVID-19 sequelae has also disrupted people’s work and daily lives (Yong, 2021). In the post-pandemic era, the main challenge for clinicians is early risk stratification and subsequent individualized treatment of COVID-19 patients.

Since the outbreak of the pandemic, considerable scientific effort has been devoted to identifying risk factors and elucidating the complex immune response associated with the clinical course and outcomes of COVID-19 (Johnston et al., 2022). Hyaluronic acid (HA) is a glycosaminoglycan with multiple important roles in cellular metabolism, water homeostasis, inflammation and immune reaction (Garantziotis et al., 2016). Recent studies showed that HA was associated with SARS-CoV-2 infection (Shi et al., 2020). This was first reported by Hellman et al. (2020), who found that accumulated HA was present in the lungs of deceased COVID-19 patients. Subsequent experimental investigation has further confirmed this discovery. Chinese researchers have identified five identical sequences shared by SARS-CoV-2 and humans that promote the accumulation of HA by upregulating HA synthase gene expression, resulting in the formation of ground-glass lesions (Li et al., 2022; Yang et al., 2022). In addition, Barnes et al. (2023) speculated that HA may play a potential role in long COVID and COVID-associated fibrosis.

Research on the clinical and prognostic implications of HA in patients with diverse experiences of COVID-19 infection remains limited. The primary objective of this study was to investigate the relationship between increased serum HA levels and COVID-19 mortality. The secondary objectives were to assess the incidence and clinical presentation of long COVID and its possible association with HA, and to compare the differences in outcomes between patients with first and second episodes of COVID-19.

## Materials and methods

### Patient population and study design

This study was conducted at the Beijing Ditan Hospital affiliated with Capital Medical University. Patients with SARS-CoV-2 (Omicron strain) infection from September 2023 to November 2023 were consecutively included. The time of first infection in patients with SARS-CoV-2 reinfection was after the liberation of Chinese epidemic control (7 December 2022). The entire study population had only been exposed to the Omicron strain. None of the study participants had received COVID-19 vaccine since their initial infection or convalescent plasma therapy during the infection. Exclusion criteria in this study: (1) severe non-infectious lung disease, such as pulmonary fibrosis and pulmonary edema; (2) progressive liver disease, such as cirrhosis and liver failure; (3) renal impairment (estimated glomerular filtration rate (eGFE) < 60 mL/min/1.73m^2^); (4) with other co-existing chronic viral infections, such as cytomegalovirus and acquired immunedeficiency syndrome; (5) pregnancy and breastfeeding; (6) with active or suspected malignancy or history of malignancy; (7) age ≤ 18 years. This study was approved by the Ethics Committee of the Beijing Ditan Hospital.

In addition, to compare serum HA levels in different populations, we also included 27 individuals who had acute SARS-CoV-2 infection but did not develop long COVID (full recovery from Omicron infection for more than 6 months) as healthy controls, according to the exclusion criteria above.

### Data collection

Baseline demographic characteristics including age, gender, body mass index, personal history, comorbidities and clinical course were collected. Routine laboratory tests were collected as part of the standard diagnostic procedures, including white blood cell, lymphocyte, hemoglobin, platelet, platelet distribution width, alanine aminotransferase, aspartate aminotransferase, direct bilirubin, albumin, glucose, creatinine, eGFR, prothrombin time, activated partial thromboplastin time, fibrinogen, thrombin time, D-dimer, CD4 count, CD8 count, C-reactive protein, erythrocyte sedimentation rate, interleukin 6. Chest CT imaging results were also collected. In addition, we investigated the incidence and common clinical manifestations of long COVID in these patients during a median follow-up of 4 months after recovery.

Reinfection is defined as a positive SARS-CoV-2 PCR test 60 days or more after a previous positive test (Eythorsson et al., 2022).

Long COVID is defined as a condition occurring 3 months after the onset of acute SARS-CoV-2 infection, with persistent clinical symptoms and disorders that last for at least 2 months and cannot be explained by any other diagnosis (Soriano et al., 2022). Typical clinical manifestations of long COVID include fatigue, shortness of breath, and cognitive dysfunction, but also others. Symptoms may be new onset after initial recovery from an acute SARS-CoV-2 episode or persist from the initial disease (Srikanth et al., 2023; Thaweethai et al., 2023).

### HA measurements

Blood samples were taken from patients on admission and serum HA concentrations were measured using a biochemical detector (Hitachi 7,020, China). We obtained two measurements and analyzed them using their mean values.

### Statistical analysis

#### Propensity score matching analysis

As previously reported, propensity score matching analysis can balance the treatment and control groups based on baseline covariates to reduce inherent selection bias and control for potential confounders (Chen et al., 2022). Thus, the propensity score was calculated using *a priori* logistic regression model based on covariates including age, sex and clinical complications. These covariates are shown in Table 1. SARS-CoV-2 reinfections were matched in a 1:2 ratio with primary infections. After propensity score matching analysis, 105 observations (93 in the first infection group and 12 in the reinfection group) were trimmed from the lower and upper tails of the propensity score due to a lack of common support. Propensity score matching was performed using an SPSS-R plugin for R packages (MatchIt, Ritools, and cem).

**Table 1:** Baseline characteristics.

Characteristics	Unadjusted			After propensity score matching		
	First infection (*n* = 182)	Reinfection (*n* = 62)	*p*	First infection (*n* = 89)	Reinfection (*n* = 50)	*p*
Personal history						
Age, (years)	68.2 ± 13.8	51.4 ± 21.1	<0.001	61.0 ± 15.4	58.6 ± 20.1	0.474
Female, *n* (%)	88 (48.3)	30 (48.4)	0.963	36 (40.4)	19 (38.0)	0.796
BMI, (kg/m^2^)	23.9 ± 4.0	23.5 ± 3.7	0.430	23.7 ± 4.1	23.7 ± 3.4	0.982
Smoking, *n* (%)	67 (36.8)	23 (37.0)	0.936	32 (36.0)	19 (38.0)	0.863
Drinking, *n* (%)	61 (33.5)	21 (33.8)	0.923	31 (34.8)	18 (36.0)	0.950
Comorbidities, *n* (%)						
Hypertension	96 (52.7)	12 (19.4)	<0.001	26 (29.2)	12 (24.0)	0.493
Diabetes mellitus	70 (38.4)	10 (16.1)	0.003	19 (21.3)	10 (20.0)	0.742
Liver disease	24 (13.2)	10 (16.1)	0.707	14 (15.7)	8 (16.0)	0.950
Cerebrovascular disease	38 (20.9)	2 (3.2)	0.003	5 (5.6)	2 (4.8)	0.880
Coronary artery disease	54 (29.6)	6 (9.7)	0.004	13 (14.6)	6 (12.0)	0.657

Values are number (percentage) or mean ± standard deviation. BMI, body mass index.

#### Comparison of variables and construction of model

Continuous variables were expressed as mean ± standard deviation or median (interquartile range) in case of skewed distribution. Differences between groups were analyzed by Student’s t test or Mann–Whitney U test. Categorical variables were presented as percentages (%) and their statistical analysis was performed by the Chi-square test or Fisher’s exact test. Univariate and multivariate regression analysis was performed using binary logistic models to assess the relationship between HA and COVID-19, and the results were presented as odds ratio (OR) and 95% confidence interval (CI). Three logistic regression models were fitted. In model 1 (the crude model), non-covariates were adjusted; in model 2, age and gender were adjusted; and in model 3, a total of 8 covariant variables were adjusted. Correlation analysis was performed using Spearman’s rank correlation coefficient and presented in a correlation matrix. The predictive power of the variables for fatal outcomes was evaluated by receiver operating characteristic (ROC) curve analysis, and their areas under the curve (AUC) were compared using a nonparametric approach. We used restricted cubic spline (RCS) to explore the relationship between HA and death risk. Statistical analysis was performed with SPSS (version 26.0), and figures were generated using R (version 4.1.3). Statistical significance was defined as a two-sided *p* value less than 0.05.

## Results

### Baseline characteristics

Baseline characteristics before and after propensity score matching are shown in Table 1. A total of 50 COVID-19 reinfections were matched to 89 primary infections. In the matched arms, the average age was approximately 60 years and most were male. Some of the patients had chronic comorbidities, such as hypertension and diabetes mellitus.

Supplementary Table S1 displays the laboratory characteristics in the reinfection and primary infection groups. Patients with repeated SARS-CoV-2 infection had higher albumin levels (*p* < 0.05). Serum levels of C-reactive protein [37.5 (9.8, 118.0) vs. 13.5 (6.9, 33.5), *p* = 0.005] and erythrocyte sedimentation rate [38.0 (15.0, 63.0) vs. 22.0 (13.0, 38.5), *p* = 0.021] were lower in the reinfection group than in the first infection group. The two groups were similar in blood routine variables, coagulation indexes and lymphocyte subsets (*p* > 0.05).

### COVID-19 imaging and clinical manifestations in the matched cohort

The results of chest CT imaging showed bilateral lung infiltration in most first infections (90.0% vs. 74.0%, *p* = 0.017). In terms of infection severity, the reinfection group had more mild cases and less respiratory failure cases (*p* < 0.05). Reinfection was associated with shorter viral clearance time [10.0 (7.0, 13.0) vs. 7.0 (5.0, 14.0), *p* = 0.026] and duration of symptoms [14.5 (9.0, 19.0) vs. 9.5 (4.0, 15.3), *p* = 0.005]. Although there was no statistical difference in the incidence of tracheal cannula, acute respiratory distress syndrome, multiple organ failure and death, the number was higher in the primary infection group than in the reinfection group, as shown in Table 2.

**Table 2:** Imaging and clinical manifestations of SARS-CoV-2 infection during hospitalization.

Characteristics	First infection (*n* = 89)	Reinfection (*n* = 50)	OR (95%CI)	*p*
Vaccine status, n (%)				
< 2 doses	36 (40.4)	13 (26.0)	1.25 (0.96–1.63)	0.120
≥2 doses	53 (59.5)	37 (74.0)	–	–
Chest CT signs, n (%)				
Bilateral lung infiltration	80 (90.0)	37 (74.0)	1.93 (1.21–3.09)	0.017
Pulmonary exudates	7 (7.9)	3 (6.0)	1.39 (0.42–2.85)	0.206
Ground-glass opacity	46 (51.6)	20 (40.0)	1.33 (0.81–2.18)	0.260
Pleural effusion	17 (19.1)	5 (10.0)	1.75 (0.71–4.29)	0.179
Pulmonary fibrosis	6 (6.7)	0	1.60 (1.39–1.86)	0.157
Clinical diagnosis, *n* (%)				
Mild	13 (14.6)	19 (38.0)	–	0.013
Moderate	48 (53.9)	17 (34.0)	–	
Severe	28 (31.4)	14 (28.0)	–	
Extrapulmonary injuries, *n* (%)				
Heart injury	8 (9.0)	1 (2.0)	3.04 (0.48–19.30)	0.255
Liver injury	12 (13.5)	4 (8.0)	1.64 (0.59–4.56)	0.371
Intestinal dysbacteriosis	12 (13.5)	2 (4.0)	2.51 (0.64–8.37)	0.206
Coagulopathy	10 (11.2)	5 (10.0)	1.10 (0.47–2.54)	1.000
Clinical course, (days)				
Viral clearance time	10.0 (7.0, 13.0)	7.0 (5.0, 14.0)	–	0.026
Duration of symptoms	14.5 (9.0, 19.0)	9.5 (4.0, 15.3)	–	0.005
Outcomes, n (%)				
Respiratory failure	19 (21.3)	2 (4.0)	3.87 (0.97–13.87)	0.016
Tracheal cannula	5 (5.6)	0	1.60 (1.39–1.85)	0.295
Acute respiratory distress syndrome	5 (5.6)	0	1.60 (1.39–1.85)	0.295
Septic shock	2 (2.2)	1 (2.0)	1.09 (0.22–5.49)	1.000
Multiple organ failure	4 (4.5)	0	1.59 (1.38–1.83)	0.552
Death	10 (11.2)	1 (2.0)	4.21 (0.64–27.64)	0.097

Values are number (percentage) or median (interquartile range). SARS-CoV-2, severe acute respiratory syndrome coronavirus-2; OR, odds ratio; CI, confidence interval.

### Levels of HA in each group

**Figure 1:**
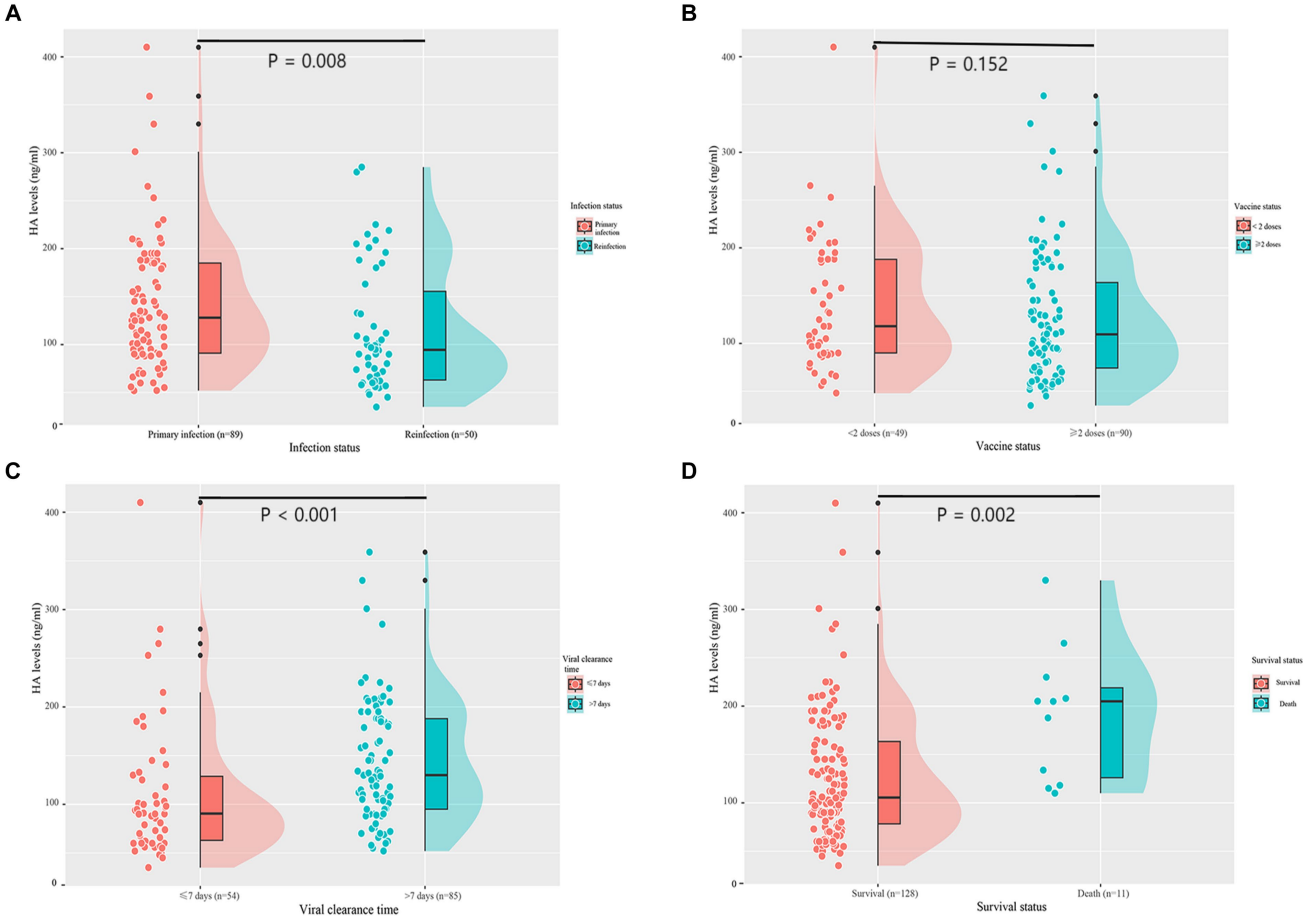
Serum HA levels according to infection status **(A)**, vaccine status **(B)**, viral clearance time **(C)**, and survival status **(D)**. HA, hyaluronic acid.

Serum HA levels were significantly lower in the reinfection group than in the first infection group [128.0 (90.5, 185.0) vs. 94.5 (62.0, 167.3), *p* = 0.008] (Figure 1A). To assess whether COVID-19 vaccination has an effect on HA expression, we divided patients into two groups according to the vaccination status: < 2 doses and ≥ 2 doses. However, there was no difference in HA levels between the two groups [118.0 (89.5, 191.5) vs. 109.5 (73.8, 168.5), *p* = 0.152] (Figure 1B). In addition, HA levels were significantly higher in patients with a viral clearance time of more than 7 days than in those with a shorter time (90.5 (61.5, 130.8) vs. 130.0 (95.0, 188.0), *p* < 0.001) (Figure 1C). As shown in Figure 1D, deceased patients had higher HA levels than survivors [105.5 (76.8, 164.5) vs. 188.0 (118.0, 208.0), *p* = 0.002]. We also analyzed HA levels in 27 healthy individuals. As expected, healthy controls had significantly lower HA levels than age- and gender-matched first infections [51.0 (39.5, 58.6) vs. 108.0 (75.0, 165.0), *p* < 0.001] and age- and gender-matched reinfections [51.0 (39.5, 58.6) vs. 86.0 (60.0, 109.0), *p* < 0.001] (Supplementary Figure S1).

### Correlation analysis of serum HA levels in COVID-19 patients

**Figure 2:**
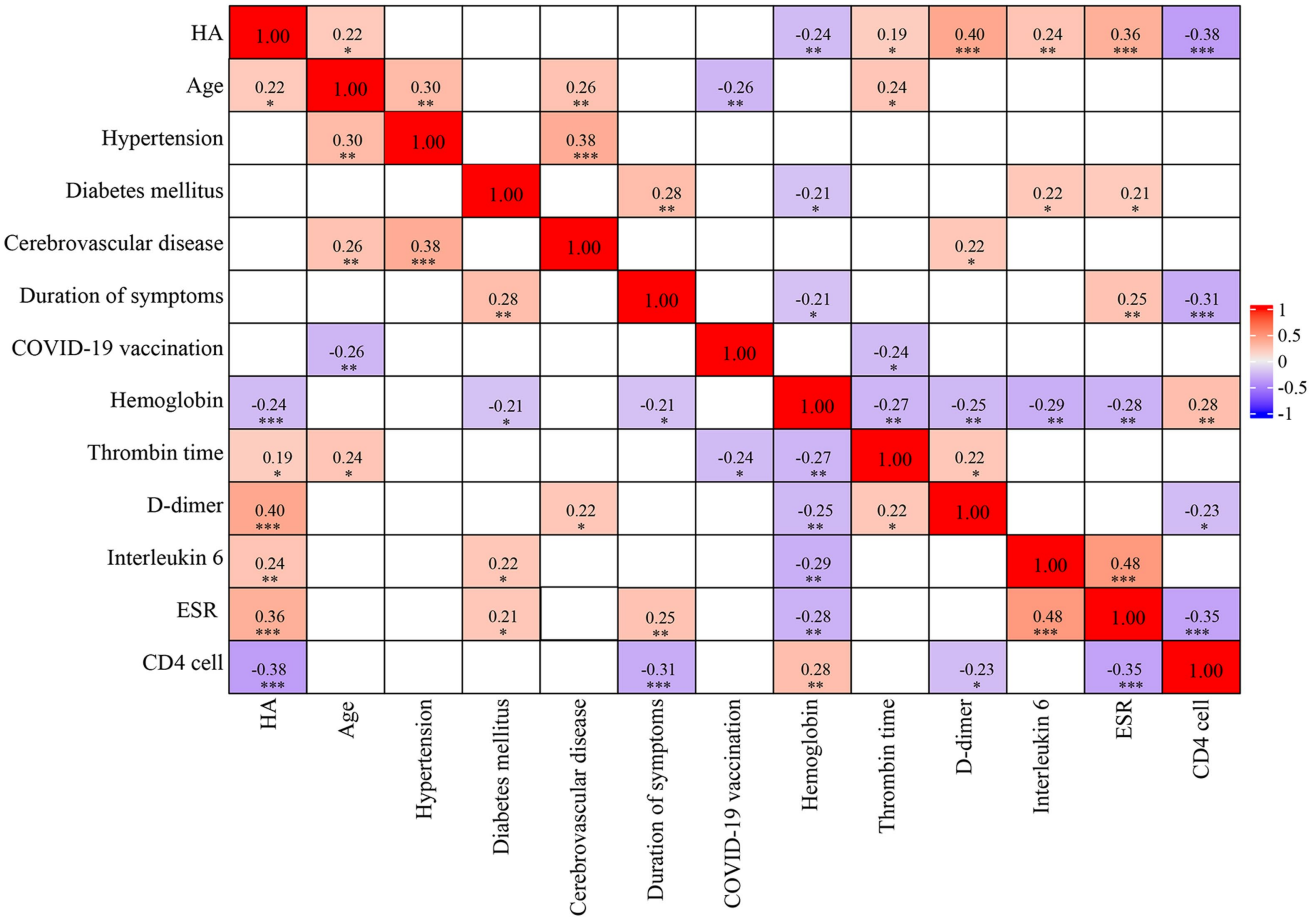
Spearman’s correlation matrix. The strength of the correlation between two variables is indicated by the color at the intersection of these variables. Colors range from dark blue (strong negative correlation; *r* = −1.0) to red (strong positive correlation; *r* = 1.0). Results are not displayed when *p* > 0.05. HA, hyaluronic acid; ESR, erythrocyte sedimentation rate.

We investigated the potential relationship between serum HA concentrations and several clinical and laboratory indicators in patients with COVID-19, as shown in Figure 2. Serum HA levels were positively associated with age (*r* = 0.22, *p* < 0.05), thrombin time (*r* = 0.19, *p* < 0.05), D-dimer (*r* = 0.40, *p* < 0.001), interleukin 6 (*r* = 0.24, *p* < 0.01) and erythrocyte sedimentation rate (*r* = 0.36, *p* < 0.001). A significant negative correlation was observed with hemoglobin (*r* = −0.24, *p* < 0.01) and CD4 count (*r* = −0.38, *p* < 0.001). However, other clinical parameters, such as hypertension, diabetes mellitus, cerebrovascular disease, duration of symptoms and COVID-19 vaccination, were not correlated with HA levels.

### Influencing factors of mortality in the matched cohort

After a univariate regression analysis, we observed that cerebrovascular disease, thrombin time, CD4 count, erythrocyte sedimentation rate and HA were associated with the risk of death (*p* < 0.05). When variables with *p* < 0.1 were included in the multivariate logistic regression analysis, lower CD4 count (OR = 6.393, 95%CI = 1.186–34.461, *p* = 0.031) and higher HA (OR = 5.435, 95% CI = 1.205–24.510, *p* = 0.028) remained statistically significant (Table 3).

**Table 3:** Influencing factors of death in the matched cohort.

Variable	N (*n* = 139)	Univariate analysis		Multivariate analysis	
		OR (95% CI)	*p*	OR (95% CI)	*p*
Age > 65 years	50	1.722 (0.438–6.775)	0.437		
Female	55	0.409 (0.081–2.063)	0.279		
Hypertension	38	3.704 (0.927–14.800)	0.064	4.586 (0.541–38.883)	0.163
Diabetes mellitus	29	1.104 (0.214–5.690)	0.906		
Liver disease	22	2.844 (0.645–12.546)	0.168		
Cerebrovascular disease	7	7.357 (1.143–47.357)	0.036	6.319 (0.265–150.434)	0.254
Cardiovascular disease	19	0.767 (0.089–6.577)	0.809		
Fully vaccinated/booster doses	90	1.101 (0.261–4.655)	0.895		
White blood cell >10 × 10^9^/L	23	1.513 (0.289–7.914)	0.624		
Hemoglobin <120 g/L	49	4.114 (0.971–17.427)	0.055	1.526 (0.206–11.281)	0.679
Platelet <100 × 10^9^/L	14	1.091 (0.125–9.559)	0.937		
Platelet distribution width > 17%	15	1.966 (0.383–10.094)	0.428		
Fibrinogen >400 mg/dL	67	2.280 (0.542–9.594)	0.261		
Thrombin time > 17 s	13	6.187 (1.298–29.503)	0.022	13.198 (1.082–160.598)	0.068
D-dimer >0.5 mg/L	82	6.267 (0.757–51.877)	0.089	2.154 (0.123–37.744)	0.599
CD4 < 500cells/ul	44	8.983 (1.765–45.717)	0.008	6.393 (1.186–34.461)	0.031
CD4/CD8 ratio < 1.4	85	5.587 (0.675–46.280)	0.111		
ESR >40 mm/h	54	6.355 (1.257–32.132)	0.025	7.377 (0.650–82.789)	0.107
Interleukin 6 > 20 pg/mL	79	6.759 (0.817–55.934)	0.076	10.937 (0.609–196.477)	0.105
HA >200 ng/mL	23	6.278 (1.705–23.111)	0.006	5.435 (1.205–24.510)	0.028

OR, odds ratio; CI, confidence interval; ESR, erythrocyte sedimentation rate; HA, hyaluronic acid.

Next, we explored the risk factors for severe infection in patients with first infection and reinfection, respectively. The results showed that HA remained an independent predictor of severe infection in both the first infection and reinfection groups (*p* < 0.05) (Supplementary Tables S2, S3).

### Association between HA and death risk

**Figure 3:**
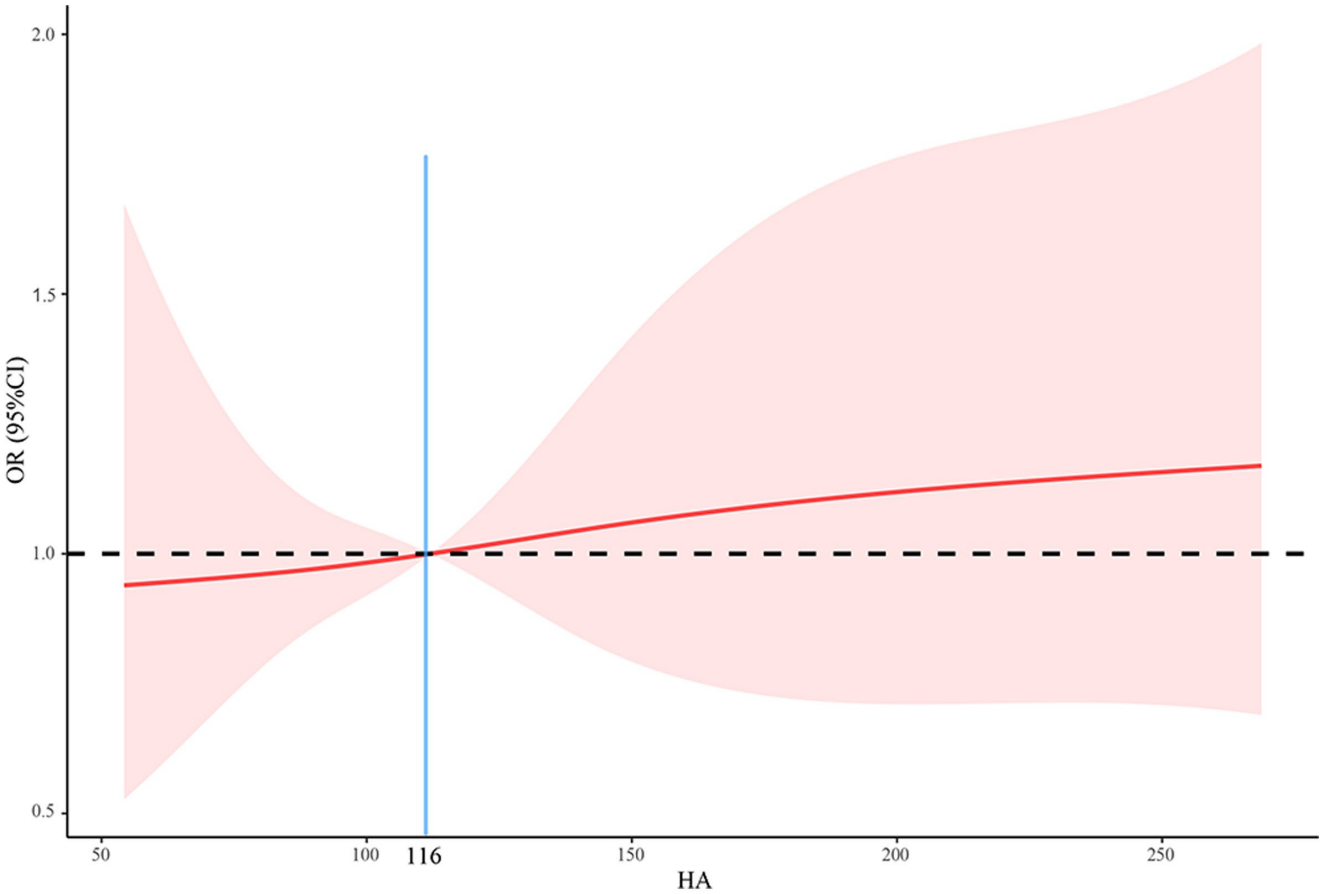
The relationship between HA and mortality using a restricted cubic spline regression model. HA, hyaluronic acid; OR, odds ratio; CI, confidence interval.

As shown in Table 4, there was a positive relationship between HA levels and the risk of death after adjustment for all covariates (OR = 1.007, 95%CI = 1.004–1.011, *p* = 0.039). In other words, higher HA levels were associated with a higher risk of death. We found a significant increase in mortality when HA levels exceeded 116 ng/mL (Figure 3). Further, we transformed HA levels from a continuous variable to a categorical variable for analysis to explore whether this association was stable. Compared to patients with HA levels less than 116, those with HA levels greater than 200 had a 10.805-fold increased risk of death (OR = 11.805, 95%CI = 1.718–117.912, *p* = 0.036; all P for trend <0.05).

**Table 4:** Association of HA with mortality in COVID-19 patients.

	Crude Model	*p*	Model 1	*p*	Model 2	*p*
	OR (95%CI)		OR (95%CI)		OR (95%CI)	
HA	1.010 (1.002–1.019)	0.012	1.011 (1.003–1.020)	0.013	1.007 (1.004–1.011)	0.039
HA groups						
<116 ng/mL	Reference		Reference		Reference	
116-200 ng/mL	4.971 (0.498–49.671)	0.172	5.283 (0.517–54.028)	0.161	3.424 (0.326–36.011)	0.305
>200 ng/mL	20.714 (2.239–191.668)	0.008	18.912 (2.015–177.528)	0.010	11.805 (1.718–117.912)	0.036
P for Trend		0.004		0.005		0.023

Crudel model: non-covariates were adjusted. Model 1: age and gender were adjusted. Model 2: age, gender, hypertension, hemoglobin, D-dimer, erythrocyte sedimentation rate, CD4 cell and interleukin 6 were adjusted. HA, hyaluronic acid; COVID-19, coronavirus disease 2019; OR, odds ratio; CI, confidence interval.

**Figure 4:**
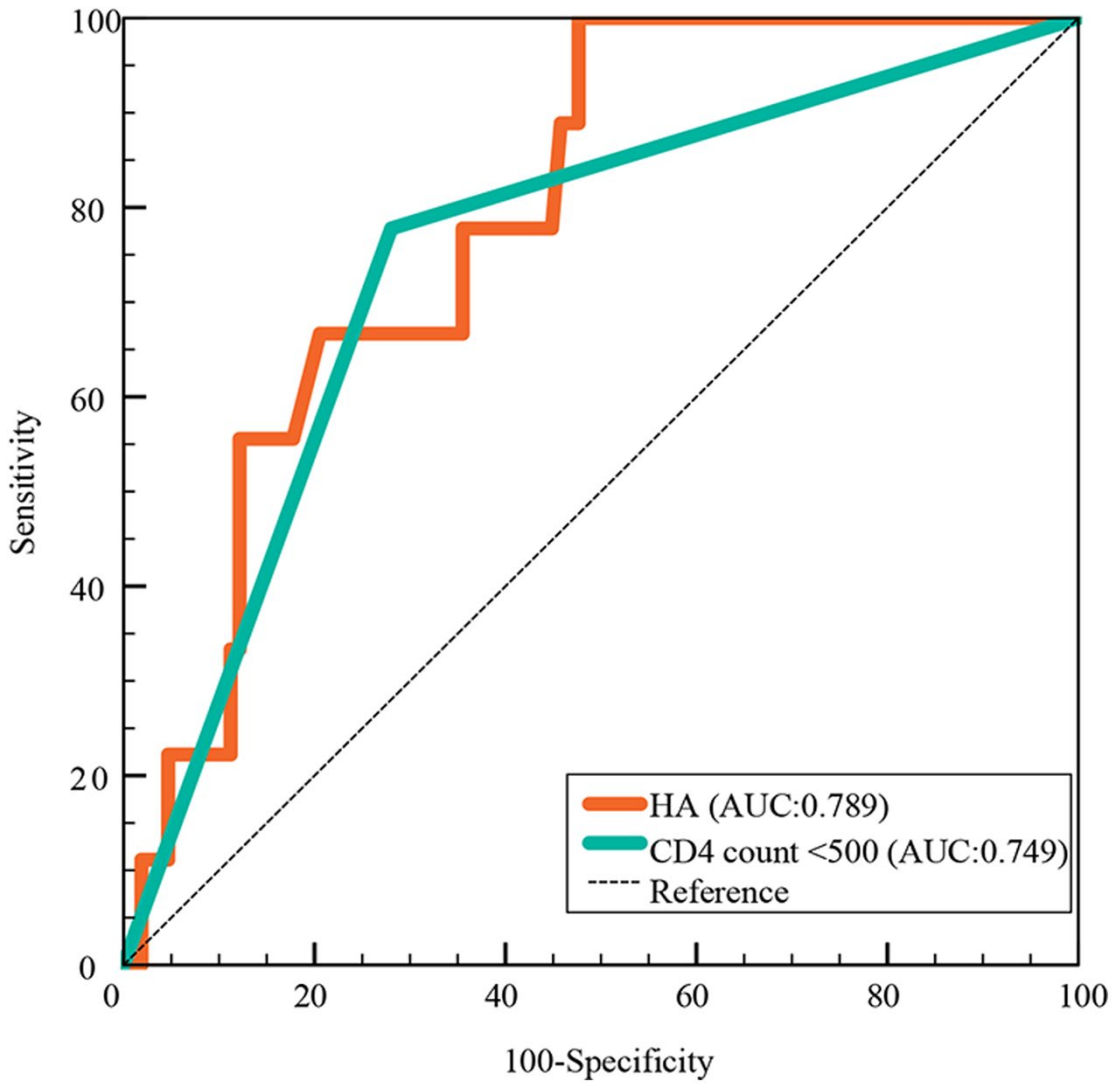
Predictive value of HA for death in hospitalized COVID-19 patients. HA, hyaluronic acid; AUC, areas under the curve.

In the ROC curve analysis, HA (AUC: 0.789) had a higher predictive value for death than CD count (AUC: 749), as shown in Figure 4.

### Relationship between long COVID and HA

In the matched cohort, 11 hospitalized patients died of severe COVID-19. We investigated the information on long COVID in the remaining 128 patients 4 months after discharge. A total of 38 (29.7%) participants self-reported having long COVID symptoms. Among patients with long COVID, the majority perceived the symptoms to be mild and did not interfere with their daily activities and lives. Only 9 patients sought medical help for long COVID symptoms. The most common symptom of long COVID was fatigue, followed by cough. Although there was no significant difference in the incidence of long COVID between the two groups, the high HA group outnumbered the low HA group (26.1% vs. 33.9%, *p* = 0.335). Although the two groups showed a similar incidence of common symptoms, such as fatigue, cough and memory decline, the low HA group tended to have a relatively lower proportion (Table 5).

**Table 5:** Self-reported long COVID symptoms and features between different HA groups.

Characteristics	Total (*n* = 128)	HA ≤ 116 ng/mL (*n* = 69)	HA > 116 ng/mL (*n* = 59)	*p*
Number of long COVID, *n* (%)	38 (29.7)	18 (26.1)	20 (33.9)	0.335
Common long COVID symptoms, *n* (%)				
Fatigue	27 (21.1)	12 (17.4)	15 (25.4)	0.267
Cough	15 (11.7)	7 (10.1)	8 (13.6)	0.549
Chest tightness/shortness of breath	14 (10.9)	6 (8.7)	8 (13.6)	0.379
Memory decline	6 (4.7)	2 (2.9)	4 (6.8)	0.300
Headache	3 (2.8)	2 (2.9)	1 (1.4)	0.469
Alopecia	1 (0.9)	1 (1.4)	0	0.353
Sweating	2 (1.9)	1 (1.4)	1 (1.4)	1.000
Insomnia	7 (5.4)	3 (4.3)	4 (6.8)	0.546
Appetite reduction	3 (2.8)	1 (1.4)	2 (3.4)	0.469
Pharyngeal discomfort	2 (1.9)	2 (2.9)	0	0.187
Seeking medical help, *n* (%)	9 (7.0)	4 (5.8)	5 (8.5)	0.555

Values are number (percentage); COVID, coronavirus disease 2019; HA, hyaluronic acid.

To further clarify whether there was an association between HA and long COVID, we performed logistic regression analysis. However, the results indicated that white blood cell count and pulmonary ground-glass opacity during acute infection were independent risk factors for long COVID rather than HA (Table 6).

**Table 6:** Risk factors associated with long COVID during follow-up.

Variable	Univariate analysis		Multivariate analysis	
	OR (95% CI)	*p*	OR (95% CI)	*p*
Age > 65 years	1.105 (0.478–2.547)	0.817		
Female	1.071 (0.418–2.254)	0.846		
Hypertension	1.932 (0.774–4.823)	0.159		
Diabetes mellitus	2.386 (0.905–6.292)	0.079	1.790 (0.620–5.171)	0.282
Liver disease	0.750 (0.222–2.350)	0.643		
Cerebrovascular disease	0.774 (0.077–7.737)	0.828		
Cardiovascular disease	0.831 (0.244–2.837)	0.768		
Fully vaccinated/booster doses	0.731 (0.311–1.715)	0.471		
White blood cell >10 × 10^9^/L	4.416 (1.501–12.988)	0.007	4.032 (1.331–12.212)	0.014
ESR >40 mm/h	1.368 (0.583–3.212)	0.471		
Interleukin 6 > 20 pg/mL	0.786 (0.343–1.801)	0.569		
HA >116 ng/mL	1.273 (0.555–2.917)	0.569		
Reinfection	1.148 (0.493–2.678)	0.749		
Bilateral lung infiltration	0.825 (0.280–2.434)	0.728		
Respiratory failure	4.096 (1.070–15.683)	0.040	1.919 (0.393–9.381)	0.421
Ground-glass opacity	3.150 (1.224–8.106)	0.017	2.866 (1.073–7.650)	0.036

COVID, coronavirus disease 2019; OR, odds ratio; CI, confidence interval; ESR: erythrocyte sedimentation rate; HA, hyaluronic acid.

## Discussion

In this prospective study of hospitalized patients with COVID-19, we found that: approximately 25.4% of patients experienced SARS-CoV-2 reinfection (Table 1); reinfection had a better clinical course and prognosis than the first infection (Table 2); HA levels were significantly higher in patients with primary infection, prolonged viral clearance time and fatal outcomes, but lower in healthy controls (Figure 1 and Supplementary Figure S1); HA was an independent predictor of death (AUC: 0.789), the risk of death increased significantly when the HA level was above 116 ng/mL (Table 3; Figures 3, 4); (5) HA levels may not be associated with long COVID (Tables 5, 6).

Although various prevention and control strategies have been implemented worldwide, including the development of vaccines and drugs and the promotion of preventive measures, the mutations of SARS-CoV-2 have inevitably led to infection and reinfection, and the death toll continues to rise (Ao et al., 2023; Cohen and Pulliam, 2023). Ongoing transmission of COVID-19 places a heavy burden on the healthcare systems in the post-pandemic era. Therefore, finding a simple and convenient predictor of poor outcome to aid early risk stratification and clinical decision making is critical. HA is a natural component of the extracellular matrix, which is synthesized by HA synthase (Garantziotis, 2022). Growing evidence suggests that HA plays a crucial role in lung pathophysiology, especially in the processes of inflammation and fibrosis (Allegra et al., 2012). At the beginning of the COVID-19 epidemic, HA was found to be abundant in the lungs and respiratory secretions of patients with severe infection (Hellman et al., 2020; Kratochvil et al., 2020). Subsequent studies found a human identical sequence in SARS-CoV-2 genome, the key therapeutic target of SARS-CoV-2, which can promote HA accumulation and ground-glass opacity formation by upregulating HA synthase expression (Li et al., 2022; Yang et al., 2022). However, due to the small sample size and limited number of relevant studies, the association between HA and adverse prognosis in the post-pandemic era needs further investigation.

Our study found that the incidence of reinfection among hospitalized patients was as high as 25.4% (Table 1). However, the rate of reinfection may vary considerably across study contexts and time periods. There was an 11.5% reinfection rate in Iceland (Eythorsson et al., 2022). Keeling (2023) predicted that the Omicron wave (2021 to 2022) was related to 18% (16–20%) of cases being reinfections in England. Currently, the global COVID-19 pandemic is dominated by the Omicron strain, which makes reinfection more common due to its high capacity for immune escape (Dhama et al., 2023). Similar to the previous study, we found that reinfection tended to be less risky than initial infection (COVID-19 Forecasting Team, 2023). Patients with second episodes of SARS-CoV-2 infection had a better clinical course, including less bilateral lung infiltration and respiratory failure, lower inflammatory factors levels, and shorter viral clearance time and duration of symptoms (Table 2). A survey from the United States also confirmed that reinfections usually featured a faster clearance time and milder type of symptoms compared to first infections (Kissler et al., 2023). Immunity from prior SARS-CoV-2 infection may be an important contributor to the reduced incidence and severity of symptomatic COVID-19 disease (Eythorsson et al., 2022). However, as this acquired protection may diminish over time and vary with viral mutations, some of the patients remain at risk of unfavorable outcomes, particularly those who experienced a severe first COVID-19 infection (Bhattacharya et al., 2022).

Previous studies have indicated the potential long-term effects of COVID-19 on the immune system and the body’s ability to produce HA (Barnes et al., 2023; Willyard, 2023). Our study showed that serum HA levels were significantly lower in the reinfection group than in the matched first infection group (Supplementary Table S1 and Figure 1). This may be due to the presence of hybrid immunity from COVID-19 vaccination and previous infection (Nordström et al., 2022). Cross-protective immunity has been demonstrated to be linked with higher neutralizing antibody titers and cellular immune responses, suggesting that it may provide a rapid and effective response to SARS-CoV-2 reinfection (Ferreira et al., 2022; Flacco et al., 2022). On the one hand, the vigilant immune system may prevent the activation of HA synthase by accelerating viral clearance; on the other hand, it may indirectly reduce serum HA levels by inhibiting the inflammatory cascade response (Solera et al., 2024). We also observed that higher HA levels were associated with prolonged viral RNA shedding time (Figure 1), making it a valuable risk factor to consider. In patients with prolonged viral clearance time, the mechanism behind the increased HA levels may be the significant activation of HA synthase by high SARS-CoV-2 viral loads (Xu et al., 2020; Li et al., 2022). Interestingly, there was no significant difference in serum HA levels between unvaccinated/partially vaccinated patients and fully vaccinated/boosted patients (Figure 1). In other words, COVID-19 vaccination did not appear to have a significant effect on HA levels. Given the impact of vaccination on the immune response and the development of novel vaccine technologies, it is necessary to further evaluate their potential role in HA production and other important physiological processes (Pandher and Bilszta, 2023).

In this study, we provide evidence that serum HA levels were significantly higher in deceased COVID-19 patients than in survivors (Figure 1). Both HA and CD count were independent predictors of COVID-19 mortality after multivariate logistic regression analysis (Table 3), but the predictive ability of HA was superior to the latter (AUC: 0.789 vs. 0.749) (Figure 4). In addition, white blood cell count and HA were independently associated with the severity of first infection (Supplementary Table S2). D-dimer and HA were independently associated with the severity of reinfection (Supplementary Table S3). Previous studies have reported that several other biomarkers could indicate disease severity and mortality in COVID-19, including inflammatory markers (erythrocyte sedimentation rate and interleukin 6), coagulation indicators (fibrinogen and D-dimer), and routine blood variables (platelet count and platelet distribution width) (Ponti et al., 2020; Semiz, 2022; Ligi et al., 2023). Although our results suggested that most of these biomarkers were positively correlated with serum HA levels, they were not independent predictors of mortality (Figure 2 and Table 3). The inconsistent results may be explained by the different study contexts. Most of these studies were conducted during the COVID-19 pandemic, when severe infections were prevalent due to the high pathogenicity of the early strains (Berlin et al., 2020). More importantly, complex interactions between inflammation and immune system, rather than the virus itself, may be responsible for the abnormalities in these hematological parameters (Yong et al., 2023). Conversely, human identical sequence in SARS-CoV-2 genome could directly upregulate HA expression by activating HA synthase (Li et al., 2022). Therefore, HA has a more accurate and specific identification ability than other biomarkers for early risk stratification in COVID-19.

Serum HA levels have been found to be elevated in patients with high cardiovascular risk, such as atheromatosis and metabolic syndrome (Papanastasopoulou et al., 2017). In our study, some of the participants had chronic complications, including hypertension, coronary artery disease and diabetes mellitus (Table 1). Our correlation analysis also showed that serum HA levels were significantly associated with age, thrombin time, D-dimer, interleukin 6, erythrocyte sedimentation rate, hemoglobin and CD4 count (Figure 2). These are potential confounders affecting both serum HA expression and COVID-19 outcome. However, HA remained independently associated with mortality after adjustment for multiple variables (Table 3). The fact that the association of HA with death was independent of these confounding factors suggests that the observed relationship of HA with fatal outcomes is not an epiphenomenon and that serum HA may serve as an independent predictor of survival in COVID-19 patients. Furthermore, we recommend that clinicians be aware of the potential risk of death when HA levels exceed 116 ng/mL. Apart from COVID-19, HA has demonstrated diagnostic significance in other respiratory disorders. A study of chronic obstructive pulmonary disease showed that the circulating HA levels were positively associated with the disease severity (Papakonstantinou et al., 2019). Moreover, the high concentrations of HA not only predict patient prognosis, but could be used as a predictor of 28-day mortality in patients with community-acquired pneumonia and acute respiratory distress syndrome (Sauer et al., 2022). Overall, we can conclude that HA may be a promising prognostic biomarker in the adverse prognosis of lung infections.

As an important component of the extracellular matrix, HA plays a facilitating role in organ fibrosis (Neuman et al., 2016). A recent review summarized the role of HA in the pathogenesis of acute and post-acute COVID-19 infection, and speculated that HA may be involved in the development of long COVID and COVID-related fibrosis (Barnes et al., 2023). However, this speculation has not yet been confirmed by clinical studies. The present study investigated the possible association between HA and long COVID. Our results indicated that the incidence of long COVID was similar between different HA level groups, and that HA was not an independent risk factor for the development of long COVID (Tables 5, 6). It must be emphasized that the small sample size may lead to some random effects and false negative results. In addition, the continuing decline in viral pathogenicity and the presence of cross-protective immunity contribute to the low incidence of severe lung lesions and long COVID (Dangi et al., 2021). Because only a small subset of patients underwent chest CT scans during follow-up, we were unable to fully assess the potential relationship between COVID-19 related pulmonary fibrosis and HA. Relying on self-reported long COVID diagnosis may affect the accuracy of the study results (Tsuzuki et al., 2022). Notably, this study only investigated the incidence and common clinical manifestations of long COVID during a median follow-up of 4 months after discharge. With increasing evidence 3 years after the pandemic, researchers have confirmed the persistence of post-COVID symptoms in 2-year follow-up after initial infection (Rahmati et al., 2023). A recent meta-analysis reported that 30% of COVID-19 patients still had psychological, neurological or physical post-COVID sequelae 2-years after SARS-CoV-2 infection (Fernandez-de-Las-Peñas et al., 2024). However, most of the studies in this review included patients infected during the earlier waves of the pandemic associated with the historical strains, and infected with other variants such as Alpha and Delta. No current long-term data is available for the Omicron variant, the most prevalent and dominant variant. In this scenario, most studies have not evaluated the effect of vaccination, antiviral drugs and reinfection on COVID-19. Given the important role of HA in SARS-CoV-2 infection and its biological properties, future studies with long-term follow-up need to investigate the possible association of HA with long COVID, especially in patients with Omicron infection.

This study has several limitations. First, we included a small sample size of 139 matched patients, which may limit the statistical validity of the study. Current COVID-19 infections are generally capable of spontaneous recovery, with only a small number of elderly or frail individuals requiring medical care. This means that the number of participants meeting our inclusion criteria is relatively small. However, the use of propensity score matching analysis increased the comparability of the variables and the credibility of the results, and may compensate for the suboptimal statistical power of the small sample size. Second, our study may underestimate the incidence of reinfection since asymptomatic individuals do not seek medical assistance. In addition, we mainly measured HA concentrations at admission, and dynamic changes during hospitalization and follow-up were unclear. Finally, this study only collected information on long COVID at a median of 4 months after discharge, the long-term association between HA and long COVID is unclear. Considering the limitations identified in the current study, further large-scale studies are needed to better understand the pathophysiological relationship between HA and COVID-19, and to determine the diagnostic value of HA for long COVID in long-term follow-up.

## Conclusion

We presented real-world data showing that lower serum HA levels in COVID-19 reinfection are associated with a better clinical course and prognosis. Serum HA may serve as a predictor of COVID-19 mortality. In addition, the risk of death increases significantly when HA levels exceed 116 ng/mL on admission. This should be taken into account when determining which patients will benefit from intensive medical care. However, HA may not be associated with long COVID. Given the ongoing COVID-19 pandemic, future studies are needed to better understand the potential effects of HA on SARS-CoV-2 infection.

## Data availability statement

The original contributions presented in the study are included in the article/Supplementary material, further inquiries can be directed to the corresponding author.

## Ethics statement

The studies involving humans were approved by Institutional Review Board of Beijing Ditan Hospital. The studies were conducted in accordance with the local legislation and institutional requirements. The human samples used in this study were acquired from All patients would underwent routine hematological tests upon admission. Therefore, we collected the remaining serum from these patients to detect hyaluronic acid levels. Written informed consent for participation was not required from the participants or the participants' legal guardians/next of kin in accordance with the national legislation and institutional requirements.

## Author contributions

YL: Writing – review & editing, Writing – original draft, Methodology, Investigation, Formal analysis. MH: Writing – review & editing, Investigation. XL: Writing – review & editing, Validation, Supervision, Resources.

## Supplementary material

SUPPLEMENTARY FIGURE S1 Serum HA levels between healthy controls and age- and gender-matched first infections and age- and gender-matched reinfections. HA, hyaluronic acid.
