# Supplementary figures and images for: Clinical and prognostic implications of hyaluronic acid in patients with COVID-19 reinfection and first infection
Source: Front Microbiol. 2024 May 31;15:1406581. doi: 10.3389/fmicb.2024.1406581 (PMC11178136; doi:10.3389/fmicb.2024.1406581)

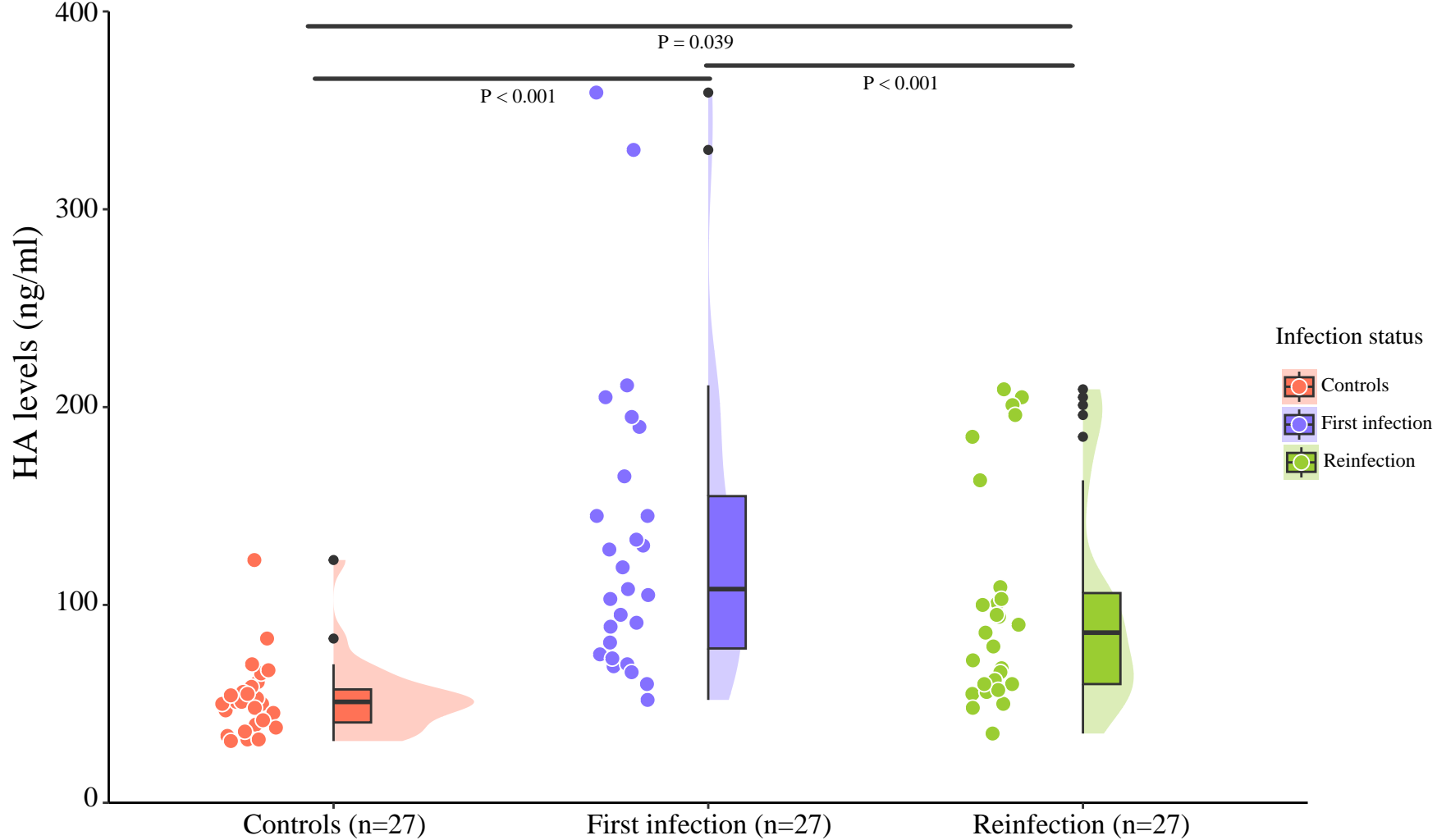

Supplement: SUPPLEMENTARY FIGURE S1 — Serum HA levels between healthy controls and age- and gender-matched first infections and age- and gender-matched reinfections. HA, hyaluronic acid. [file Data_Sheet_1.PDF]
