# Supplementary material for: Clinical and prognostic implications of hyaluronic acid in patients with COVID-19 reinfection and first infection
Source: Front Microbiol. 2024 May 31;15:1406581. doi: 10.3389/fmicb.2024.1406581 (PMC11178136; doi:10.3389/fmicb.2024.1406581)
Supplement: Supplementary file 2 [file Presentation_1.pdf]

Supplementary Table 1 Comparison of the laboratory characteristics in the matched cohort

Characteristics	First infection (n = 89)	Reinfection (n = 50)	P
Biochemical Parameters			
Alanine aminotransferase, (U/L)	21.0 (13.8, 32.3)	18.5 (13.0, 38.5)	0.886
Aspartate aminotransferase, (U/L)	26.0 (17.8, 36.8)	21.0 (16.0, 32.0)	0.155
Direct bilirubin, (μmol/L)	3.7 (2.5, 6.0)	3.8 (2.5, 5.0)	0.405
Albumin, (g/L)	37.0 (32.0, 41.0)	41.0 (35.8, 44.0)	0.016
Glucose, (mmol/L)	6.5 (5.7, 8.8)	6.9 (5.8, 9.0)	0.599
Creatinine, (μmol/L)	73.0 (57.8, 91.5)	67.0 (53.0, 85.5)	0.367
eGFR, (ml/min/1.73m^2)	93.3 ± 22.8	87.6 ± 29.7	0.142
Blood routine variables			
White blood cell, (×10^9/L)	6.6 ± 3.2	7.0 ± 2.6	0.512
Lymphocyte, (×10^9/L)	0.8 (0.5, 1.2)	1.0 (0.7, 1.3)	0.079
Hemoglobin, (g/L)	125.5 ± 25.1	124.4 ± 29.1	0.827
Platelet, (×10^9/L)	177.9 ± 69.0	183.0 ± 56.2	0.685
Platelet distribution width, (%)	15.43 ± 1.22	15.63 ± 1.16	0.137
Coagulation indexes			
Prothrombin time, (s)	12.0 (11.0, 13.0)	12.0 (11.0, 13.0)	0.394
APTT, (s)	32.0 (30.0, 34.3)	31.0 (30.0, 33.0)	0.366
Fibrinogen, (mg/dL)	430.0 (337.5, 485.3)	368.5 (328.8,444.0)	0.093
Thrombin time, (s)	14.3 (13.0, 15.7)	14.0 (13.0,15.6)	0.490
D-dimer, (mg/L)	0.6 (0.4, 1.1)	0.5 (0.3, 1.0)	0.190
Lymphocyte subsets			
CD4, (cells/ul)	283.5 (209.5, 502.0)	336.5 (174.5, 531.3)	0.360
CD8, (cells/ul)	255.0 (155.8, 373.3)	251.0 (176.3, 403.5)	0.560
Inflammatory indicators			
C-reactive protein, (mg/L)	37.5 (9.8, 118.0)	13.5 (6.9, 33.5)	0.005
ESR, (mm/h)	38.0 (15.0, 63.0)	22.0 (13.0, 38.5)	0.021
Interleukin 6, (pg/mL)	24.5 (7.8, 55.8)	21.0 (10.0, 61.5)	0.789
HA, (ng/mL)	128.0 (90.5, 185.0)	94.5 (62.0, 167.3)	0.008

Abbreviations: Values are median (interquartile range) or mean ± standard deviation. eGFR, estimated glomerular filtration rate; APTT, activated partial thromboplastin time; ESR, erythrocyte sedimentation rate; HA, hyaluronic acid.

Supplementary Table 2 Risk factors for severity of COVID-19 in first infections

Variable	Univariate analysis		Multivariate analysis	
	OR (95% CI)	P	OR (95% CI)	P
Age	1.004 (0.972-1.037)	0.803		
Female	0.636 (0.230-1.758)	0.383		
Hypertension	3.000 (1.055-8.532)	0.039	2.186 (0.570-8.379)	0.254
Diabetes mellitus	2.625 (0.842-8.180)	0.096	2.633 (0.572-12.115)	0.214
Liver disease	0.651 (0.159-2.662)	0.550		
Cerebrovascular disease	0.681 (0.067-6.914)	0.745		
Cardiovascular disease	0.174 (0.021-1.447)	0.106		
Fully vaccinated/booster doses	1.207 (0.444-3.278)	0.712		
White blood cell >10×10^9/L	3.020 (0.886-10.286)	0.077	3.965 (0.998-15.755)	0.045
Hemoglobin <120g/L	2.408 (0.867-6.691)	0.092	1.202 (0.249-5.792)	0.819
Platelet <100×10^9/L	0.414 (0.082-2.087)	0.285		
Platelet distribution width >17%	0.818 (0.147-4.557)	0.819		
Fibrinogen >400mg/dL	3.250 (1.106-9.548)	0.032	3.442 (0.662-17.894)	0.142
Thrombin time >17s	1.286 (0.281-5.891)	0.746		
D-dimer >0.5mg/L	2.357 (0.801-6.938)	0.120		
CD4 <500cells/ul	4.964 (1.734-14.210)	0.003	2.714 (0.739-9.970)	0.132
CD4/CD8 ratio <1.4	1.704 (0.618-4.700)	0.303		
ESR >40mm/h	5.824 (1.951-17.384)	0.002	0.893 (0.181-4.394)	0.889
Interleukin 6 >20pg/mL	1.100 (0.411-2.946)	0.850		
HA	1.017 (1.007-1.027)	0.001	1.017 (1.007-1.028)	0.001

Abbreviations: COVID-19, coronavirus disease 2019; OR, odds ratio; CI, confidence interval; ESR: erythrocyte sedimentation rate; HA, hyaluronic acid.

Supplementary Table 3 Risk factors for severity of COVID-19 in reinfections

Variable	Univariate analysis		Multivariate analysis	
	OR (95% CI)	P	OR (95% CI)	P
Age	1.076 (1.024-1.130)	0.004	1.024 (0.958-1.094)	0.485
Female	0.229 (0.043-1.225)	0.085	0.045 (0.002-1.319)	0.072
Hypertension	3.571 (0.800-19.547)	0.095	8.994 (0.918-25.095)	0.070
Diabetes mellitus	1.667 (0.329-8.434)	0.537		
Liver disease	2.167 (0.405-11.601)	0.366		
Cerebrovascular disease	0.608 (0.265-5.901)	0.689		
Cardiovascular disease	4.667 (0.670-32.488)	0.120		
Fully vaccinated/booster doses	0.350 (0.082-1.500)	0.157		
White blood cell >10×10^{9}/L	0.455 (0.047-4.361)	0.494		
Hemoglobin <120g/L	1.727 (0.446-6.686)	0.429		
Platelet <100×10^{9}/L	0.618 (0.185-2.102)	0.189		
Platelet distribution width >17%	0.591 (0.059-5.905)	0.654		
Fibrinogen >400mg/dL	0.576 (0.128-2.588)	0.472		
Thrombin time >17s	1.186 (0.185-3.890)	0.869		
D-dimer >0.5mg/L	9.000 (2.153-16.938)	0.008	12.487 (1.185-23.539)	0.036
CD4 <500cells/ul	2.347 (0.564-9.766)	0.241		
CD4/CD8 ratio <1.4	3.333 (0.618-17.970)	0.161		
ESR >40mm/h	0.550 (0.098-3.073)	0.496		
Interleukin 6 >20pg/mL	1.750 (0.432-7.084)	0.433		
HA	1.019 (1.006-1.031)	0.003	1.020 (1.004-1.037)	0.015

Abbreviations: COVID-19, coronavirus disease 2019; OR, odds ratio; CI, confidence interval; ESR: erythrocyte sedimentation rate; HA, hyaluronic acid.
